# Comparing the effects of various β-blockers on cardiovascular mortality in breast cancer patients

**DOI:** 10.1186/s40959-024-00217-1

**Published:** 2024-03-26

**Authors:** Mantasha Tabassum, Soumya G. Chikermane, Camille Johnson, Noor M. Abdulkareem, Elisabeth M. Wang, Michael L. Johnson, Meghana V. Trivedi

**Affiliations:** 1 Department of Pharmacological and Pharmaceutical Sciences, University of Houston College of Pharmacy, 4349 Martin Luther King Blvd, 77204 Houston, TX USA; 2 Department of Pharmaceutical Health Outcomes and Policy, University of Houston College of Pharmacy, Houston, TX USA; 3 Department of Pharmacy Practice and Translational Research, University of Houston College of Pharmacy, Houston, TX USA

**Keywords:** Cardiovascular mortality, Breast cancer, β-blockers, Metoprolol, Atenolol, Carvedilol, Heart failure

## Abstract

**Background:**

Cardiovascular (CV) disease is a leading cause of death in breast cancer (BC) patients due to the increased age and treatments. While individual β-blockers have been investigated to manage CV complications, various β-blockers have not been compared for their effects on CV death in this population. We aimed to compare CV mortality in older BC patients taking one of the commonly used β-blockers.

**Methods:**

This retrospective cohort study was conducted using the Surveillance, Epidemiology and End Results (SEER) - Medicare data (2010–2015). Patients of age 66 years or older at BC diagnosis receiving metoprolol, atenolol, or carvedilol monotherapy were included. The competing risk regression model was used to determine the risk of CV mortality in the three β-blocker groups. The multivariable model was adjusted for demographic and clinical covariates. The adjusted hazard ratio (HR) and 95% confidence intervals (CI) were reported for the risk of CV mortality.

**Results:**

The study cohort included 6,540 patients of which 55% were metoprolol users, 30% were atenolol users, and 15% were carvedilol users. Metoprolol was associated with a 37% reduced risk of CV mortality (*P* = 0.03) compared to carvedilol after adjusting for the covariates (HR = 0.63; 95% CI 0.41–0.96). No significant difference in the risk of CV mortality between atenolol and carvedilol users was observed (HR = 0.74; 95% CI 0.44–1.22).

**Conclusions:**

Our findings suggest that metoprolol is associated with a reduced risk of CV mortality in BC patients. Future studies are needed to confirm these findings and understand the mechanism of action.

## Background

Cardiovascular (CV) mortality poses a major threat in survival of patients with breast cancer (BC) even though cancer-specific survival has improved since 1990 due to enhanced screening and effective therapies [1–4]. CV diseases account for up to 1.9 times higher risk of death in BC patients than the general population [1]. The risk for CV mortality in BC is higher with increasing age at diagnosis, with women who are 65 years of age or older being the most vulnerable [3–7]. The increased risk of CV mortality in long-term BC survivors stems from cardiotoxic chemotherapies, radiotherapy, and immunotherapies [1, 5, 8–11]. Cytotoxic and targeted chemotherapies including anthracyclines, cyclophosphamide, and trastuzumab targeting human epidermal growth factor receptor 2 (HER2) account for 27% of cardiac dysfunctions, 19% of which is New York Heart Association (NYHA) class III/IV in BC patients [12–15]. In general, anti-HER2 drugs have a higher incidence of cardiotoxicity, accounting for 0.5 to 3.9% [16, 17]. Radiotherapy has been a known factor for inducing dose-dependent cardiotoxicity which accounts for 1.76-fold higher risk of cardiac mortality than patients not exposed to radiation [8, 11, 18]. Immune checkpoint inhibitors, which are being used in BC patients only recently, have a rare (< 1%) but significant risk of severe myocarditis with a mortality rate of up to 50% [18, 19]. The major CV adverse effects of these therapies are ischemic heart disease, heart failure, as well as cardiac dysrhythmias [3, 6, 12, 20, 21].

Several pharmacologic strategies are used to reduce the disease progression and CV mortality in non-cancer patients with heart failure with a reduced ejection fraction (HFrEF). These strategies include the usage of β-blockers, angiotensin-converting enzyme inhibitors (ACEi), and angiotensin receptor blockers (ARBs) [22]). These agents have also been investigated prospectively for their effects on left ventricular ejection fraction (LVEF) as a surrogate of CV incidences with the objective of mitigating or reducing CV toxicities associated with anti-HER2 agents and anthracyclines in BC patients [23–33]. Among β-blockers, nebivolol [24] and bisoprolol (with or without lisinopril) [25, 26] significantly attenuated anthracycline-containing chemotherapy- or trastuzumab-induced LVEF decline compared to control/placebo group. While carvedilol [23, 27–30] had mixed data, metoprolol [31–33] did not have a significant effect on LVEF decline associated with anthracycline-containing chemotherapy. Despite the promising results of some studies, a major limitation has been smaller sample size, with most enrolling less than 100 patients per study arm. Additionally, previous studies have evaluated a surrogate endpoint with LVEF but there is a lack of data evaluating outcomes with β-blockers and CV mortality in this patient population. The objective of this study was to compare three commonly used β-blockers (metoprolol, atenolol, and carvedilol) for their effects on CV-related mortality in BC patients using the Surveillance, Epidemiology, and End Results (SEER) - Medicare dataset.

## Methods

### Data sources

This was an administrative claims and registry-based retrospective cohort study conducted using the SEER - Medicare data from 2010 to 2015. This data links the SEER registry information from seventeen states to Medicare utilization claims data for patients diagnosed with cancer [34].

### Study population

**Fig. 1:**
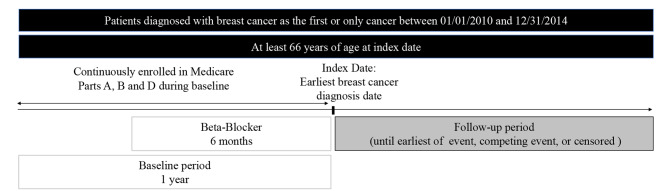
Study design

Patients who were diagnosed with breast cancer as their first cancer if they had more than one cancer diagnoses or only cancer between 01/01/2010 and 12/31/2014 were identified using International Classification of Diseases for Oncology, third edition (ICD-O-3 codes) (C500-C509). Those 66 years of age or older at BC diagnosis were included in this study because the SEER - Medicare data primarily includes patients older than 65 [35]. The earliest date of BC diagnosis was termed as the index date, and patients had to be continuously enrolled in Medicare parts A, B and D and not enrolled in Part C or Health Maintenance Oranizations (HMOs) during the 1-year baseline period before the index date. Patients receiving the three most commonly used β-blockers: metoprolol or atenolol or carvedilol monotherapy, for at least 6-months before the index date were included and β-blocker monotherapy was the exposure of interest, with these groups being mutually exclusive. The outcome was CV mortality and was defined using the vital status recode variable (value = dead) and the cause of death to site recode variable (value= ‘50060’ indicating death due to diseases of the heart), from the Patient Entitlement and Diagnosis Summary File. Sociodemographic and clinical characteristics were based on the Andersen Behavioral model and measured during the baseline period. The study design is described in Fig. 1.

### Statistical analysis

Descriptive characteristics were calculated to show the difference in sociodemographic and clinical characteristics across the cohorts. Means and standard deviation was reported for continuous variables, and frequency and percentages were reported for binary or categorical variables. The Fine and Grey subdistribution hazard model was used to assess the risk of CV mortality associated with the three β-blocker groups, accounting for the competing risk of death due to other reasons. We also tested to ensure the hazard of the CV mortality was proportional over time. Patients were followed until the occurrence of CV mortality (event of interest), or until they were censored. Patients were censored if they were lost to follow-up, switched β-blockers for any reason, discontinued the β-blocker for any reason, or at end of follow-up (12/31/2015). Patients were considered lost to follow-up at the point when they were no longer enrolled in Medicare Parts A, B, and D during the follow-up period. The multivariable model was adjusted for age at BC diagnosis, sex, race-ethnicity, stage and subtype of BC, Charlson comorbidity index (CCI, calculated at baseline), statin use, and indicator for multiple cancers. The adjusted hazard ratio (HR) and 95% confidence intervals (CI) were reported for the risk of CV-related mortality for metoprolol and atenolol compared to carvedilol.

## Results

### Patient demographics and clinical characteristics

The study cohort included 6,540 BC patients diagnosed between 2010 and 2014, of which 3,622 (55.38%) were metoprolol users, 1,929 (29.49%) were atenolol users, and 989 (15.12%) were carvedilol users. Table 1 summarizes demographic and clinical characteristics of patients in the three cohorts. Most patients in each cohort were older than 75 years of age (56.25%), White (86.82%), and female (99.19%). A majority of the patients had BC as the only cancer (87.81%) and were diagnosed at stage 0 or 1 (55.64%). Stage 0 refers to in situ tumors where cancer cells have not spread out of the ductal or lobular structure.

### CV mortality among β-blockers

In the three β-blocker groups, 1.9% (*n* = 70) of patients using metoprolol, 1.9% (*n* = 36) of patients using atenolol, and 4.0% (*n* = 40) of patients using carvedilol died due to CV-related events. Median time to CV-related mortality was 528 days in the metoprolol group, 411 days in the atenolol group, and 356 days in the carvedilol group.

### Adjusted CV mortality among β-blockers

Table 2 displays the multivariable competing risk model assessing the CV-related mortality risk in the 3 groups after adjusting for patient demographics like age at BC diagnosis, sex, race-ethnicity, stage and subtype of BC, CCI, statin use, and indicator for multiple cancers. Metoprolol was associated with a 37% reduced risk of CV-related mortality [adjusted HR (95% CI): 0.63 (0.41 to 0.96), *P* = 0.03] compared to carvedilol. There was no significant difference in the risk of CV-related mortality found between atenolol compared to carvedilol users [adjusted HR (95% CI): 0.74 (0.44 to 1.22)].

**Table 1:** Baseline demographics and clinical characteristics of patients by different β-Blocker usage

Variable	Metoprolol *N* = 3622	Atenolol *N* = 1929	Carvedilol *N* = 989	Total *N* = 6540	*P*-value
	N (%)	N (%)	N (%)	N (%)	
**DEMOGRAPHIC VARIABLES**					
**Age at index, in years**					< 0.0001
65–70	689 (19.02)	465 (24.10)	165 (16.68)	1319 (20.17)	
71–75	870 (24.02)	456 (23.64)	216 (21.84)	1542 (23.58)	
> 75	2063 (56.96)	1008 (52.26)	608 (61.48)	3679 (56.25)	
**Race**					< 0.0001
White	3177 (87.71)	1685 (87.35)	816 (82.51)	5678 (86.82)	
Black	252 (6.96)	108 (5.60)	110 (11.12)	470 (7.19)	
Other	171 (4.72)	122 (6.32)	*	352 (5.38)	
Unknown	*	*	*	40 (0.61)	
**Hispanic or Latino**	158 (4.36)	87 (4.51)	75 (7.58)	320 (4.89)	0.0001
**Gender**					0.7066
**Female**	3595 (99.25)	*	*	6487 (99.19)	
**Male**	27 (0.75)	*	*	53 (0.81)	
**CLINICAL VARIABLES**:					
**Charlson comorbidity Index**					< 0.0001
0	1429 (39.45)	959 (49.72)	209 (21.13)	2597 (39.71)	
1–3	1869 (51.60)	870 (45.10)	573 (57.94)	3312 (50.64)	
4 or greater	324 (8.95)	100 (5.18)	207 (20.93)	631 (9.65)	
**Statin use**					< 0.0001
**Yes**	2163 (59.72)	1104 (57.23)	671 (67.85)	3938 (60.21)	
**No**	1459 (40.28)	825 (42.77)	318 (32.15)	2602 (39.79)	
**Breast Cancer stage**					< 0.0001
**0**	493 (13.61)	291 (15.09)	119 (12.03)	903 (13.81)	
**I**	1532 (42.30)	853 (44.22)	351 (35.49)	2736 (41.83)	
**II**	928 (25.62)	484 (25.09)	312 (31.55)	1724 (26.36)	
**III**	274 (7.56)	151 (7.83)	98 (9.91)	523 (8.00)	
**IV**	180 (4.97)	75 (3.89)	51 (5.16)	306 (4.68)	
**Unknown**	215 (5.94)	75 (3.89)	58 (5.86)	348 (5.32)	
**Diagnosed at breast cancer stage 0 or 1**					< 0.0001
**Yes**	2025 (55.91)	1144 (59.31)	470 (47.52)	3639 (55.64)	
**No**	1597 (44.09)	785 (40.69)	519 (52.48)	2901 (44.36)	
**Breast Cancer subtypes**					0.7382
HR-/HER2+	193 (5.33)	101 (5.24)	44 (4.45)	338 (5.17)	
HR+/HER2-	2256 (62.29)	1217 (63.09)	635 (64.21)	4108 (62.81)	
HR-/HER2-	269 (7.43)	125 (6.48)	69 (6.98)	463 (7.08)	
HR+/HER2+	219 (6.05)	114 (5.91)	67 (6.77)	400 (6.12)	
Unknown	685 (18.91)	372 (19.28)	174 (17.59)	1231(18.82)	
**Breast Cancer as the only cancer**					0.7100
**Yes**	3191 (88.10)	1685 (87.35)	867 (87.66)	5743 (87.81)	
**No**	431 (11.9)	244 (12.65)	122 (12.34)	797 (12.19)	

**Table 2:** Adjusted Risk of CV Mortality among β-blocker users in breast cancer

Adjusted Model	Reference	HR (95% CI)	*P*-value
Metoprolol	Carvedilol	0.63 (0.41–0.96)	0.03
Atenolol		0.74 (0.44–1.22)	0.24

## Discussion

In this study, we compared the incidence of CV mortality in BC patients taking the 3 most prescribed β-blockers (metoprolol, atenolol, and carvedilol) using the SEER - Medicare database from 2010 to 2015. We found that metoprolol users were less likely to experience CV death compared to carvedilol users in the adjusted model. Atenolol use did not have a significant effect on CV mortality compared to carvedilol. The novelty of our study comes from the comparison of three commonly used β-blockers for their effect on CV mortality in patients with BC. While other studies have tested and reported effects of β-blockers on LVEF decline [23–33] and on BC mortality [36–41], none of the studies have evaluated CV mortality in patients with BC. Furthermore, none of the studies in BC patients have compared different β-blockers.

In the general population without cancer, β-blockers are utilized to reduce CV mortality in a variety of disease states, including heart failure, acute myocardial infarction, and hypertension [42, 43]. Of the β-blockers evaluated in various studies, metoprolol succinate [44] and carvedilol [45–47] have shown a statistically significant decrease in mortality compared to other β-blockers or placebo in patients with HFrEF and are two of the three β-blockers recommended for management of this disease state (bisoprolol being the third) [48]. In comparison to metoprolol tartrate, carvedilol treatment results in a greater reduction in mortality when used in the treatment of HFrEF [49–52]. However, this difference seems more pronounced in men than in women [50]. No significant survival differences are reported between metoprolol and carvedilol in the treatment of acute myocardial infarction [53, 54].

The landmark trial (COMET) comparing carvedilol and metoprolol tartrate in the HFrEF population found carvedilol to be superior for reducing all deaths and CV deaths [49]. A critique of this study is that the median daily dose of metoprolol tartrate was only 85 mg daily where in the trial that found mortality benefit with metoprolol succinate, the median dose was 159 mg daily [55]. This lower daily median dose may have impacted results of COMET, but ultimately metoprolol tartrate was not found to have mortality benefit. Therefore, future studies should evaluate different salts and doses of metoprolol for the CV survival benefit in BC patients.

Both metoprolol and atenolol are second generation selective β1-blockers. Metoprolol and carvedilol are also inverse agonists and capable of inhibiting basal β receptors activity [56]. Carvedilol is a third-generation nonselective β-blocker with selective alpha 1-adrenoceptor antagonist and vasodilatory effects [57]. Both metoprolol and carvedilol are lipophilic compounds in contrast with atenolol, which is hydrophilic. Additionally, carvedilol has antioxidant properties that may contribute to the benefit it has shown in patients with HFrEF [58]. However, these differences do not explain the superiority of metoprolol in our studies. It is possible that carvedilol doses were not sufficient in our cohort as the daily doses of 12.5 mg seem to be necessary to improve LVEF in patients receiving anthracycline-containing regimen [23, 27].

There are several limitations to our study. First, we did not assess the type of metoprolol salts patients were on, as metoprolol succinate had been found to have mortality benefit in HFrEF patients and metoprolol tartrate has not in robust, randomized controlled trials. Additionally, β-blocker dosing and information of other baseline medications patients were on (i.e., ACEi/ARBs) were not extracted but could have impacted the outcome we observed. The use of CCI only to assess baseline CV disease and risk factors is another limitation. For example, the CCI does not include hypertension, which is prevalent in postmenopausal BC patients [59]. Certain BC treatments such as anthracyclines, radiation therapy, anti-HER2 agents, and other targeted therapies are associated with cardiac complications, such as cardiomyopathy [14]. These patient-, cancer-, and treatment-specific risks are also not explicitly considered in the CCI. However, with the signals identified in the current study, subequent studies can aim at addressing these limitations in future analysis. Finally, patient baseline LVEF as well as % of patients with HFrEF in different arms were not available and could have impacted results. Future studies incorporating these parameters can further validate our findings and answer the key question on the selection of β-blocker for improving CV survival in BC patients.

## Conclusions

In summary, our study is the first one comparing the effects of various β-blockers on CV mortality in BC patients and suggests superiority of metoprolol in these patients. Our findings prompt additional investigation with larger sample size to investigate the effects of doses and type of individual β-blockers in reducing CV mortality in BC patients. In addition, similar investigation on individual ACEi and ARBs on CV mortality are also needed to select appropriate agents to improve CV-specific survival in patients with BC.

## Data Availability

This study used the linked SEER-Medicare database. The interpretation and reporting of these data are the sole responsibility of the authors. The authors acknowledge the efforts of the National Cancer Institute; the Office of Research, Development and Information, CMS; Information Management Services (IMS), Inc.; and the Surveillance, Epidemiology, and End Results (SEER) Program tumor registries in the creation of the SEER-Medicare database. The data that support the findings of this study are available from IMS, but restrictions apply to the availability of these data, which were used under license for the current study, and so are not publicly available. The datasets used to conduct this study are available upon approval of a research protocol from the National Cancer Institute. Instructions for obtaining these data are available in the [SEER-Medicare] database [Unique persistent identifier, https://healthcaredelivery.cancer.gov/seermedicare/obtain/].

## List of Abbreviations

ACEi: Angiotensin-converting enzyme inhibitors
ARBs: Angiotensin receptor blockers
BC: Breast cancer
CCI: Charlson comorbidity index
CI: Confidence interval
CV: Cardiovascular
HFrEF: Heart failure with a reduced ejection fraction
HMO: Health Maintenance Oranization
HR: Hazard ratio
ICD-O-3: International Classification of Diseases for Oncology, third edition
LVEF: Left ventricular ejection fraction
NYHA: New York Heart Association
SEER: Surveillance, Epidemiology and End Results
